# ‘Almost nothing is firmly established’: A History of Heredity and Genetics in Mental Health Science

**DOI:** 10.12688/wellcomeopenres.20628.2

**Published:** 2024-08-08

**Authors:** Sarah Chaney, Sarah Marks, Rebecca Wynter

**Affiliations:** 1 Centre for the History of the Emotions, Queen Mary University of London, London, England, UK; 2 School of Historical Studies, Birkbeck University of London, London, England, UK; 3 School of Historical Studies, Universiteit van Amsterdam, Amsterdam, North Holland, The Netherlands

**Keywords:** genetics, mental health, psychiatry, history, eugenics, schizophrenia, lived experience, humanities, heredity

## Abstract

**Background:**

For more than a century, scientists have tried to find the key to causation of mental ill health in heredity and genetics. The difficulty of finding clear and actionable answers in our genes has not stopped them looking. This history offers important context to understanding mental health science today.

**Methods:**

This article explores the main themes in research on genetics and inheritance in psychiatry from the second half of the nineteenth century to the present day, to address the question: what is the history of genetics as a causative explanation in mental health science? We take a critical historical approach to the literature, interrogating primary and secondary material for the light it brings to the research question, while considering the social and historical context.

**Results:**

We begin with the statistics gathered in asylums and used to ‘prove’ the importance of heredity in mental ill health. We then move through early twentieth century Mendelian models of mental inheritance, the eugenics movement, the influence of social psychiatry, new classifications and techniques of the postwar era, the Human Genome Project and Genome Wide Association Studies (GWAS) and epigenetics. Setting these themes in historical context shows that this research was often popular because of wider social, political and cultural issues, which impacted the views of scientists just as they did those of policymakers, journalists and the general public.

**Conclusions:**

We argue that attempting to unpick this complex history is essential to the modern ethics of mental health and genetics, as well as helping to focus our efforts to better understand causation in mental ill-health.

For a succinct timeline of the history of psychiatric genetics, alongside the history of other proposed causes for mental ill-health, visit: https://historyofcauses.co.uk/

## Disclaimer

*The following review covers difficult themes including eugenics, sterilization and 'race science'. It contains offensive historical terminology and explores views that are now rightly deemed unpleasant and dangerous. Themes and language are used only to improve an understanding of how such attitudes have informed aetiology and mental health science, and how this thinking may survive through intellectual baggage and the continued use of concepts formed in the past. Terms used are distinct to historical periods. Terminology of the past does not always map neatly onto current usage (and vice versa).*

## Introduction

For over a hundred years, clinicians and scientists have attempted to find the answer to the causes of mental ill health in heredity and genetics. The inability to find clear and actionable answers in our genes has not stopped them looking. A survey and focus group carried out by the current authors in 2023 as part of a wider project on mental health causation ( https://historyofcauses.co.uk/) also showed that the idea that anxiety, depression or psychosis can be passed down through the generations is a prevalent way in which people with lived experience make sense of mental health difficulties ( Marks *et al.*, 2023). This sense of a hereditary factor was not always attributed to genetics – with concepts such as intergenerational trauma also invoked – and was rarely seen as the sole factor in causation. However, it reminds us that the concept of genetics, whether metaphorical or literal, remains powerful across a range of groups. Critical reflection on the history of these ideas in mental health science is timely.

This article explores the main themes in research on genetics and inheritance in psychiatry from the second half of the nineteenth century to the present day, to address the question: what is the history of genetics as a causative explanation in mental health science? We take a critical historical approach to the literature, interrogating primary and secondary material for the light it brings to the research question, while considering the social and historical context. Our research also indicates some of the consequences of this emphasis on heredity in causation. It focuses on Europe and North America, where there were many commonalities (but also some distinct differences) in this period. Much of this article covers schizophrenia, as the main diagnosis claimed to have a genetic origin, from the early twentieth century onwards. Anxiety and depression, by contrast, rarely feature in this history. The article makes an original contribution by being the first to synthesise the history of genetics and psychiatry up to the present day, taking into account developments since the 1990s. It sheds light on how debates within the scientific and clinical communities about the meaning and implications of genetic research have developed. Importantly, it also brings to the fore diverse perspectives from patient and lived experience communities in relation to hereditarian aetiologies. These voices, until recently, have not been well integrated into mainstream Mental Health Science.

We begin with the statistics gathered in nineteenth-century asylums and used to ‘prove’ the importance of heredity in mental ill health. We then move through early twentieth-century Mendelian models of mental inheritance, the eugenics movement, the influence of social psychiatry, new classifications and techniques of the postwar era, the Human Genome Project and Genome Wide Association Studies (GWAS) and epigenetics. Setting these themes in historical context indicates that such research was often popular because of wider social, political and cultural issues, which impacted the views of scientists just as they did those of policymakers, journalists and the general public. Attempting to unpick this complex history is essential to the modern ethics of mental health, as well as helping to focus efforts to better understand causation in mental ill-health.

For a succinct timeline of the history of psychiatric genetics, alongside the history of other proposed causes for mental ill-health, visit: https://historyofcauses.co.uk/

## Ethics and consent

This study received ethical approval from Birkbeck University of London as a routine proposal approved by the Departmental Research Ethics Officer on 06/03/23 (Ethical approval number: clt26llko0081qp4q5iz6fiig). Participants’ informed consent to share anonymous responses and quotations was given before participating in the lived experience survey and focus groups. Ethics approval did not include the sharing of raw data; however, this data does not underpin the research but instead formed part of a wider project on causation that raised some of the questions that are discussed below.

## Methods

This paper was produced from a review of published secondary and primary literature on mental health and genetics, from the mid nineteenth century to the present day, using a critical historical approach. The review addressed the question: what is the history of genetics as a causative explanation in mental health science? All literature consulted is detailed in the bibliography.

This research was explicitly historical, and much of the data was from books and edited volumes. To ensure all types of data were captured, we reviewed all material in the Wellcome Library (subject fields: Eugenics – History; Genetics, Medical; Human Genome Project; Mental Disorders – Genetics; Psychiatry - History) and Senate House Library (subject field: Mental Illness – Genetic Aspects), selecting those that explicitly dealt with heredity and mental ill-health. Keyword searches for ‘gene*’ or ‘inherit*’ and ‘psychiatr*’ or ‘mental health’ pulled up a small number of additional items that were not found by the initial shelf search, in particular primary source material from 1900 – 1960.

Archive material from the Museum of the Mind was available from a previous research project conducted by Chaney, in which she created a database of patient and clinical records from 1880 – 1900, as well as summarising all annual reports, physician’s weekly reports and patient magazines from the same era. For more recent literature on GWAS and psychiatric genomics, PubMed was searched for “Genome Wide Association Studies AND psychiatr*”, “Genome Wide Association Studies AND Schizophrenia”, “Psychiatr* AND genomics” and “Psychiatr* AND epigenetics”.

To better incorporate lived experience perspectives in the framing of the research, the authors carried out a focus group and anonymous survey with a total of 40 people with lived experience of mental ill-health in spring 2023, which helped to inform subsidiary questions explored: how have genetic approaches to mental health impacted on service users? What have been the benefits and drawbacks of this approach from a lived experience perspective? And why has the genetic explanation of mental health proved enduringly popular?

This focus group and survey was part of a wider project into the history of causes in mental ill-health. The sample size was small (7 participants in the focus group and 30 survey participants). The main objective of this focus group and survey was to consult the opinions of those with lived and living experience to ensure that the timeline and resources would be useful and relevant to them.

The aim of both the focus group and survey was consultative: to better understand how people with lived experience view their own diagnosis and what sources they use to gather further information. Focus group participants were recruited by the McPin Foundation and the Youth Advisory Group at the University of Birmingham’s Institute for Mental Health. The session was led by the existing group leader alongside two of the research team, with a small number of questions designed to encourage open responses. The survey was distributed through the National Survivor User Network (NSUN), open to the first 30 respondents fully completing the survey. A voucher in recognition of the time spent was offered to all participants.

## Heredity as a statistical category, c. 1840 - 1900

The twentieth-century scientific interest in inheritance emerged from nineteenth-century asylum psychiatry. This focus came not from biology, but from statistics. As the asylum system grew rapidly across Europe and North America during this period, many institutions began to gather figures on presumed causation ( Gausemeier, 2015; Porter, 2016). Heredity featured often, but was not paramount. In the published *Statistics of Insanity* for London’s influential Bethlem Royal Hospital from 1846-1855, ‘hereditary’ was the most common cause assigned to women (171 out of 1663 women admitted) and the second most common attributed to men (89 out of 1066) ^2^ ( Hood, 1856, pp. 52–3). However, mental illness was more often ascribed to reasons other than heredity.

This is not to say that the idea of inherited mental disorders began with statistics. In previous decades, patients admitted to private institutions were asked about any family history of mental disorder, with answers in the affirmative ‘taken as virtually explanatory’ ( Smith, 1999, p. 106). In the absence of clear record-keeping, evidence of this notion of heredity was more colloquial. However, it meant that when asylum psychiatrists (known as alienists) began to gather and analyse detailed statistics in the mid-nineteenth century, they were already inclined towards an inheritance-based model of mental ill health. At the Bethlem Royal Hospital, for example, case books from the later nineteenth century indicate that *any* positive answer to family history was recorded as heredity, no matter how vague: ‘two children died of meningitis’; ‘a sister became depressed at an advanced age owing to money troubles’; ‘an aunt is said to be "peculiar"’, ‘mother is “nervous”’; ‘Father nervous man’ ( ‘Patient Casebooks’, 1880-1900, CB-116-CB-164).

The growth of the public asylum system and its systematic record-keeping in the second half of the nineteenth century was thus an important factor in the development of heredity as a model of causation. Yet different institutions recorded heredity differently. In 1850s Bethlem, Hood had only assigned ‘hereditary’ as a cause if no other reason was listed. Some facilities only counted illness in parents, grandparents and siblings as evidence of heredity; others recorded *any* relative, no matter how distant. Psychiatrist Kenneth S. Kendler has compared four ‘typical’ studies carried out in Britain, France and Germany between 1841 and 1864. These all differed in terms of which familial relationships and traits (from diagnosed illness to undefined ‘eccentricity’) they considered proved a hereditary cause. Different criteria meant very different results, ranging from around 31% to almost half of patients assigned a ‘hereditary’ cause of illness ( Kendler, 2021). Psychiatrists in the second half of the century considered a range of ways of standardising their data to combat this issue, although these proved largely too complex to implement ( Gausemeier, 2015, pp. 472–3; Porter, 2016, p. 87).

It was, however, rare that heredity was deemed the sole cause of illness. Alienists tended to split causation into two parts: ‘predisposing’ and ‘exciting’. This dual model emerged from eighteenth-century French studies of heredity in disease ( López-Beltrán, 2007, p. 119). It was popularised in German psychiatry by Wilhelm Griesinger in the mid-nineteenth century and recorded in British asylum casebooks around the same time ( Gausemeier, 2008, p. 150). At Bethlem, for example, this model appeared on pre-printed forms from 1860 onwards. ‘Heredity’ was characterised as a predisposing cause. Specific recent occurrences – including infection, injury, disappointment in love or financial trouble – were viewed as ‘exciting’ factors that pushed a person into outright illness ( ‘Patient Casebooks’, 1860). Nonetheless, heredity was not a fixed or definite category. It often but not always included congenital conditions, whether inherited or acquired (such as syphilis contracted in the womb), as well as other disorders thought to be hereditary but later proven not to be, like tuberculosis ( López-Beltrán, 2007).

During the later decades of the century, concern over so-called ‘hereditary degeneration’ proliferated. Bénédict Morel, psychiatrist and founder of degeneration theory in France, claimed that families with hereditary ‘taint’ became increasingly physically and mentally degraded, so that by the third generation they were marked by ‘insanity’, ‘idiocy’ and visible physical decline ( Morel, 1857). Morel’s treatise was picked up across Europe ( Dowbiggin *et al.*, 1985, p. 191). In Britain, Henry Maudsley wrote characteristically bleakly that ‘no one can escape the tyranny of his organization; no one can elude the destiny that is innate in him’ ( Maudsley, 1873, p. 76). The notion of the ‘third generation’ characterised attitudes to heredity across a range of conditions, with mental illness, learning disability, epilepsy and neurological disorders all considered evidence of familial mental instability. Physician and author Arthur Conan Doyle’s short story ‘The Third Generation’ (1894), for example, subsumed congenital syphilis into general anxiety about degenerate heredity ( Doyle, 2007, pp. 30–9). In the early twentieth century, these ideas entered the courtroom, through justice Oliver Wendell Holmes’ infamous 1927 decision in the Buck v Bell case that ‘three generations of imbeciles were enough’ ( Kevles, 1993, p. 10). The case – which led to the sterilization of Carrie Buck – was used to justify sterilization in the US for decades to follow, as a genealogical turn, discussed in the following section, took hold.

This bleak view emerged despite the fact that one thing all statistical and family studies of heredity in mental health had in common was that they did not analyse the spread of mental ill health across the general population, outside institutions. It might, after all, be mere coincidence that an asylum patient’s father had also suffered from mental distress, or it might be associated entirely with shared environment or life experiences. In 1895, Jenny Koller, assistant to Swiss degeneration theorist August Forel, published the very first genealogical study of mental illness which included a ‘control’ group of healthy individuals. Koller found that 59% of her control group were ‘hereditarily burdened’, indicating that mental illness was found in *most* families, and not just a few ‘degenerate’ ones ( Gausemeier, 2008, p. 153; Porter, 2018, pp. 288–289). Yet, by this time, heredity was so firmly established as a cause of mental illness that Koller’s discovery simply redirected the search. In Germany, researchers moved towards establishing genetic links for specific diagnoses, primarily schizophrenia, inspired by influential psychiatrist Emil Kraepelin’s diagnostic taxonomy ( Porter, 2016, p. 95). In Britain and other European countries, predisposing and exciting causes continued to be emphasised through the notion that some people might be (genetically) more vulnerable to life’s shocks than others.

By the end of the nineteenth century, ‘neurotic’ or ‘nervous’ inheritance had come to characterise the idea of predisposition. These ‘persons predisposed’, British psychiatrist George Fielding Blandford explained, were ‘a large class who have inherited a tendency to the malady from parents or forefathers, and are liable to transmit it in turn to their offspring’ ( Blandford, 1892, p. 996). This ‘nervous inheritance’, Blandford claimed, was not just the result of mental illness in the family. It could also be derived from parents or grandparents with a combination of ‘epilepsy, dipsomania, hysteria, hypochondriasis, or neuralgia’ and – oddly specifically given his lack of any supporting evidence – one from this list plus an additional relative with phthisis (TB) or gout ( Blandford, 1892, p. 997). Thus, in 1880, Bethlem Hospital began to ask if any patient admitted had previously experienced ‘neuroses’ (as well as phthisis, chorea, fits or fevers) and by 1890 they had also added alcoholism, syphilis and diabetes to the list of relevant prior conditions ( ‘Patient Casebooks’, 1880, CB-116; ‘Patient Casebooks’, 1890, CB-138). Again, this list contains disorders subsequently proven to have a congenital (syphilis) or genetic (chorea in Huntington’s disease) component alongside those later found to have neither.

Concern about heredity affected patients as well as doctors. Sir Francis Norton, the eponymous ‘third generation’ in Doyle’s story, attends his doctor in part to ask about his upcoming wedding and the story ends with his sudden death, a probable suicide. It seems likely that, in real life, Doyle was consulted about marriage by his patients, as were psychiatrists in their asylum and private practices. Nancy J., a former patient, wrote to Bethlem Hospital on the occasion of her engagement in 1891 to ask ‘was my insanity of a nature that it would not be right for me to marry?’ ( ‘Patient Casebooks’, 1888, CB-135, p. 94). However, as historian Mathew Thomson concludes, before the late nineteenth century heredity was largely regarded as a personal issue, not something that was seen a reason for confinement in an institution ( Thomson, 2010, p. 117). As the twentieth century turned, anxiety about inherited mental distress became more public, associated with a new Mendelian model of inheritance in mental health.

## Genealogical models of heredity, c. 1895 - 1945

Interest in Mendelian genetics in psychiatry emerged in the 1900s. Gregor Mendel, the Austrian monk and biologist, had published his experiments with pea plants in 1865. Mendel concluded that certain characteristics (sometimes called ‘unit traits’) could be passed directly from parent plants to their children, in different proportions depending on whether a trait was dominant or recessive. It was not until the early twentieth century that these ideas were taken up in the human sciences. In 1902, British professor of biology William Bateson – who coined the term ‘genetics’ in 1905 – published a defence of Mendel’s ideas that influenced British genetics for decades to come ( Bateson & Mendel, 1902; Bateson, 1928, p. 93).

In a recent close history of the development of Mendelism, focusing on the differences between Bateson and Oxford biologist Walter Frank Raphael Weldon, historian Gregory Radick claims that our modern view of genetics was shaped by this debate ( Radick, 2023). While Bateson held up genes as the most important factor in inheritance, Weldon preferred a more complex and contextual view of Mendel’s theories. Although Weldon’s approach was shared (perhaps surprisingly) by University College London (UCL) chair of eugenics, and protégé of Francis Galton, Karl Pearson, Weldon’s untimely death saw Bateson’s fatalistic approach to genetics triumph. Yet even Bateson was not sure that his theories of heredity could be applied to mental ill-health. ‘[I]n dealing with diseases of the nervous system,’ he wrote, ‘it must be remembered that many of them depend for their appearance on the presence of external stimuli’ and the ‘descent of such peculiarities is therefore beyond the range of our analysis’ ( Bateson & Mendel, 1909, p. 229).

Nonetheless, a ‘genealogical turn’ took hold in mental health science, especially in Germany, inspired by Ottokar Lorenz’s 1898 *Handbook of Scientific Genealogy* ( Gausemeier, 2015, p. 475). While there had been some interest in family pedigrees before this time, with French, German and Austrian psychiatrists undertaking family tree research, it was not widespread – and barely present at all in Britain. Lorenz’s ‘ancestor charts’ became a popular research method in Germany, albeit with limited results ( Gausemeier, 2015, p. 476). A more extensive study was published in Sweden. In 1913, Herman Lundborg reconstructed seven generations of one family, in an effort to demonstrate that myoclonic epilepsy was inherited as a recessive Mendelian trait ( Gausemeier, 2015, p. 476). Outside Scandinavia, however, researchers were hampered by a lack of available genealogical data.

Nevertheless, in the United States, an influential group of scientists tried their hardest to prove that mental illness was inherited through a Mendelian model. Biologist Charles Davenport, who founded the Eugenics Record Office (ERO) in 1910, gathered a huge amount of data from institutions across the United States. These were the records of asylums, hospitals, prisons, almshouses, schools and refuges, of insurance companies and gymnasiums, alongside new data gathered by 257 fieldworkers in an effort to standardise diagnosis ( Porter, 2018, pp. 258-9). Davenport set up two subcommittees on mental health. One focused on ‘insanity’, headed by psychiatrist Aaron Rosanoff with fieldworkers Gertrude Cannon and Florence Orr. The other looked at ‘feeblemindedness’, and was headed by Henry Herbert Goddard (who published the influential and later infamous *The Kallikak Family* (1912) from his work). Both committees concluded that their data ‘proved’ that these conditions were inherited as Mendelian recessive traits, despite abandoning early claims to diagnosis-specific inheritance.

Both studies were criticised at the time, by statisticians and psychiatrists in Europe and the United States. As a widely-used British psychiatric textbook put it in 1927, ‘very little even of what is probable is known of the inheritance of mental instability, and almost nothing is firmly established’ ( Henderson & Gillespie, 1927, p. 25). Historian Theodore Porter points out that the data gathered by Davenport’s team was given far less weight than the idea that inspired its collection. Although the final study contained vast numbers of tables and seemingly careful coding, the conclusions scarcely changed, based firmly on Davenport’s pre-existing Mendelian theories and not on any ‘painstaking pedigrees’ ( Porter, 2018, pp. 269). Despite criticism, the ‘proof’ of direct heredity in Rosanoff’s and Goddard’s studies was held to justify an increase in sterilization and marriage laws across the United States. Like Bateson in England, who had spoken positively of the use of Mendelian laws in the pursuit of eugenics, Davenport and his colleagues were all inspired by the so-called ‘science of racial improvement’ introduced by Francis Galton. And yet, if mental illness *was* inherited as a recessive Mendelian gene as these scientists claimed, no such pursuit could actually work, since more people would be invisible carriers than outwardly affected. Historian Ian Dowbiggin thus suggests that sterilization and immigration restrictions were supported by some psychiatrists for reasons other than their heredity research: social, economic and political ( Dowbiggin, 1997, p. 233).

Rosanoff’s conclusions received their heaviest criticism in Germany, largely from a statistical perspective. Since Rosanoff looked only at ‘insane’ children born to ‘normal’ parents, critics claimed this skewed his data. If mental illness were a recessive gene, many parents carrying it would have *only* ‘normal’ children, who were missing from Rosanoff’s statistics ( Gausemeier, 2015, p. 479). This did not, however, mean that research into heredity was unconnected with eugenics in Germany—far from it. However, research tended to focus on the inheritance of specific diagnoses, rather than the blanket notion of ‘insanity’ popular elsewhere. This began an emphasis on the presumed heritability of schizophrenia that spread across Europe through the twentieth century. A focus on schizophrenia appeared to address the concern about whether mental illnesses were specific enough to be regarded as Mendelian unit traits, since Kraepelin claimed ‘dementia praecox’ (as schizophrenia was initially known) to be a biologically specific disease. Ernst Rüdin, later known as the father of psychiatric genetics, decided to determine whether this newly christened disorder followed Mendelian patterns. His results indicated that there could be no simple Mendelian explanation for its inheritance: just 5%, rather than the expected 25%, of the child generation bore the diagnosis ( Gausemeier, 2015, p. 479).

This inconvenient data did not change Rüdin’s views, a theme that recurs time and again in the history of scientific research into the heredity of mental illness. Not only did Rüdin continue to believe that mental illness (particularly schizophrenia) was inherited, but he was influential as a eugenicist and, later, a member of the Nazi Party. Thanks in no small part to Rüdin’s work, the diagnosis of schizophrenia became an especial focus for the Nazis. Rüdin was one of the ‘most ardent advocates’ of a regime of sterilization that saw more than 130,000 psychiatric patients sterilized ( Read & Masson, 2013, p. 35). Wilhelm Werner's drawings of the procedure carried out on him between 1934 and 1938, held by the Prinzhorn Collection in Heidelberg, Germany, offer a disturbing insight into the extreme violation experienced by psychiatric patients ( Prinzhorn Sammlung, 2024). In Werner’s drawings, complex machines surround the horrified patient, while a blank parade of doctors and nurses use mysterious implements on his genitals. From 1939 onwards, through the Aktion T-4 campaign, 73% of Germans with a diagnosis of schizophrenia are thought to have been murdered ( Read & Masson, 2013, p. 37; Torrey & Yolken, 2010). 

Not only was this a horrific episode in the history of psychiatry – and humanity – but the tragic irony is that it also served to disprove negative eugenics. The devastating programme even created a climate where new diagnoses flourished. As geneticist Adam Rutherford points out, while the number of people in Germany living with a diagnosis of schizophrenia was extremely low in comparison to other countries after the Second World War, the number of new diagnoses was high – more than double the rate in other European countries ( Rutherford, 2022, p. 229). Indeed, Rutherford concludes, the harsh physical and psychological environment created by Nazi genocide and the devastation of war might well have created a climate in which the ‘environmental risks for individuals to develop mental health problems’ increased ( Rutherford, 2022, p. 230).

## ‘After’ eugenics? social psychiatry, blood groups and monogenic theories of inheritance

While interest in Mendelian models of inheritance lingered after the Second World War, this was accompanied by new methods of investigating genetics ( Swanson, 2006). Historian Jenny Bangham has examined the interest in blood group analysis, following the introduction of mass blood donation in the UK in 1939. Geneticists focused this research on mental disorders, funded by the Rockefeller Foundation − whose interests in eugenics are well documented ( Kevles, 1995, p. 208) – and led by R.A. Fisher, professor of eugenics at UCL. Fisher, Lionel Penrose and John Fraser Roberts carried out blood group surveys in two psychiatric hospitals but concluded that blood studies showed a ‘genetical factor’ only among the ‘mentally deficient’ ( Bangham, 2020, pp. 56–7). Blood, however, remained central to genetics: the Human Genome Project (1990–2003), for example, relied on multiple blood donations ( *Human Genome Project Fact Sheet*, 2022). Penrose, meanwhile, made his name with work on so-called ‘mental deficiency’, which became a key area of chromosome investigation by geneticists in the post-war era ( de Chadarevian, 2020, chap. 2; Penrose, 1949).

However, it was twin studies and not blood or genealogy – or even chromosome analysis – that became seen as the key to heredity and mental health in the post-war era. Francis Galton is often credited with the introduction of this method, thanks to his attempts to use twins to decipher the ‘relative powers of nature and nurture’ in 1875 ( Galton, 1875, p. 566). The specific twin study method – of comparing identical and fraternal twins – was first proposed by Hermann Siemens, a German dermatologist, in 1924 ( Rende *et al.*, 1990). In the post-war era, geneticists tended to cite Luxenburger (1928) and Rüdin (1915) as forerunners in the field. Rosenthal claimed, in 1963, that there had been ‘no great advance in our knowledge about heredity in schizophrenia since Luxenburger’ ( Rosenthal, 1963, pp. 6–7).

Luxenberger’s method assumed that a comparison of identical twins (who have the exact same DNA) and non-identical twins (who do not) can be used to account for environmental factors in inheritance. This approach has been critiqued by those, like psychologist Jay Joseph, who question whether environment can really be assumed equal for identical and non-identical twins. Identical twins tend to spend more time together and are treated more similarly by parents, teachers and peers ( Joseph, 2006, p. 17). Adoption studies, which have attempted to fill this gap, have been similarly criticised for failing to account for patterns of adoption and the life experiences of adopted families ( Rose *et al.*, 1990). While this rejection of a genetic model of mental health has been dismissed by some scientists as overtly political, this avoids the fact that, for most of its history, the genetic explanation of mental ill health has *also* been political, embedded as it is in eugenics.

Twin studies in psychiatry measure ‘concordance’ rates – whether both of a pair of twins receive the same diagnosis in their lifetimes. In Gottesman and Shields’ 1972 schizophrenia study, for example, 50% of identical but only 9% of non-identical twins did so ( Mascie-Taylor, 1993, p. 136). Post-war twin studies were led by an interest in diagnosis. This shifted the genetics of mental health away from the ‘neurotic inheritance’ which had characterised British psychiatry in the inter-war period, towards an emphasis on schizophrenia. Just as German researchers had previously claimed, now other European scientists agreed that schizophrenia was the most discrete and specific of psychiatric diagnoses. An example of this shift is shown clearly in the work of Eliot Slater, one of the most prominent genetically oriented psychiatrists in Britain during the post-war era. Slater began his research career in 1931 at the Maudsley Hospital, where heredity was emphasised as a cause of mental illness ( Jones & Rahman, 2008, p. 112). In 1934, he received a Rockefeller scholarship to study at Ernst Rüdin’s centre in Munich. Although Slater later emphasised his dislike of Nazi policies, he nonetheless remained an advocate of eugenics – contributing to *Eugenics Review* during and after the Second World War – and continued to cite research by Rüdin and other Nazi scientists ( Slater, 1971, pp. 18–19).

While Slater’s wartime work also explored neurosis and the ‘neurotic constitution’, in the post-war era he became known for his schizophrenia studies ( Slater, 1958). This stemmed from his work on twins, funded by the Medical Research Council, and carried out from 1935 to 1953. Slater claimed that of 41 pairs of identical twins in which one was diagnosed with schizophrenia, in 28 cases the other twin was ‘likewise schizophrenic’ (68.3%). What’s more, Slater thought the concordance rate was ‘likely’ to be closer to 94.9%. Since many of his twins were under 40, he assumed that they would develop symptoms later in life ( Slater, 1971, p. 104). This idea of calculating ‘risk’ of illness, often attributed to Luxenburger, introduced yet another hypothetical into genetic research. Without detailed follow-up, how was one to determine who was ‘at risk’ of developing schizophrenia, other than by confirmation bias?

Twin studies remained embedded in a Mendelian model of genetics. Even before his twin study was complete, Slater was writing of the ‘schizophrenic gene’ in the singular ( Slater, 1971, p. 83). The same theory of a disease-specific gene was advanced in the US from 1936 by German émigré Franz Kallmann, a student of Rüdin. Kallmann held that schizophrenia was caused by a recessive gene – and, in a rare mention of another diagnosis, manic depression by a dominant one ( Kevles, 1995, p. 207). Kallmann had Jewish heritage and had fled Germany, at risk of falling victim to Nazi eugenic policies. He nonetheless argued that eugenics in the form of sterilization was appropriate for people diagnosed with schizophrenia *and* their relatives ( Read & Masson, 2013, p. 39). While Lionel Penrose and others found Kallmann’s twin studies ‘unconvincing’ at the time, his work was still being cited in many textbooks in 2008 and 2009 ( Kevles, 1995, p. 207; Read & Masson, 2013, p. 39).

Eliot Slater, meanwhile, was a hugely influential voice in British psychiatry. In 1959, he founded the Psychiatric Genetics Research Unit at London's Maudsley Hospital – the global hub for psychiatric genetics ( Harper, 2008, p. 337) – and was its director until his retirement ten years later. In 1961, he became editor of the ‘ailing’ *Journal of Mental Science* and, by 1962, had turned it into the *British Journal of Psychiatry*, with a marked emphasis on biological and genetic approaches to aetiology ( Slater, 1971, p. xvi). As his career came to an end, Slater remained positive that schizophrenia was caused by a single gene, despite admitting that there could be no simplistic dominant or recessive Mendelian model of inheritance, since two parents who both had a diagnosis of schizophrenia were able to produce children without ( Slater & Cowie, 1971, p. 71).

## The Genain Quadruplets: polygenic theories of inheritance and gene-environment interaction (1960s – 1980s)

By the 1960s, however, a monogenic theory of schizophrenia appeared overly simplistic to most researchers; while his twin study work remained influential, Slater was in the minority. American psychologist David Rosenthal, who recalled turning to Slater’s work on genetics when he began his study into the ‘Genain’ quadruplets in 1955, soon diverged from this monogenic model ( Slater, 1971, p. xx). So did other twin theorists like Gottesman and Shields, who developed a polygenic theory in the 1960s ( Gottesman & Shields, 1967).

Rosenthal’s study is often cited as a central case in the shift from psychological to genetic theories of causation in mental illness in general, and schizophrenia in particular. While this paper has made clear that there is a much longer history and range of models within genetics and causation, the Genain study was nonetheless influential. In the United States, it offered a route away from psychoanalytic models of mental illness that had dominated for much of the twentieth century. For three years, from 1955-8, Rosenthal and his colleagues studied four sisters – identical quadruplets – who had moved into the National Institute of Mental Health for the purpose of investigation. All four young women had been diagnosed with schizophrenia, and were thus deemed the perfect case to investigate the potential genetic origins of the disorder.

The study was published in 1963 and, despite the assertions later made about it, was quite guarded in its claims. Rosenthal adopted the carefully sceptical view that ‘we still do not know if it [schizophrenia] is really a unitary disorder’ ( Rosenthal, 1963, p. 6). He pointed out that the quadruplets had such an ‘extraordinary’ family history that their similar diagnoses were not conclusive proof of a genetic aetiology for schizophrenia ( Rosenthal, 1963, p. 7). Isolated from their peers and dominated by an abusive father, the quads experienced a unique and unusual upbringing, as described by Audrey Clare Farley ( Farley, 2023). While psychologists like Richard Bentall have blamed Rosenthal for the late twentieth century obsession with a genetic origin for schizophrenia, the original Genain study was much more carefully worded ( Bentall, 2009). Indeed, Rosenthal explicitly stated that his ‘position is one which considers both genetic and environmental factors important in such disorders’ ( Rosenthal, 1963, p. 574). He went on to present several models for an interaction between heredity and environment, something he noted had been little investigated: variance analysis, activation, augmentation, facilitation-resistance and reciprocal escalation ( Rosenthal, 1963, pp. 575–9). It does not appear that these were taken up seriously by others.

Despite David Rosenthal’s interest in gene-environment interaction, most subsequent researchers tended to emphasise the genetic elements of the Genain study, as well as the adoption studies carried out by Rosenthal and colleagues in the 1960s. Of course, this was influenced by the way the topic was presented: the pseudonym for the quadruplets (Genain) came from the Greek for ‘dire birth’ or ‘dreadful gene’, emphasising one cause over the many others outlined ( Rosenthal, 1963, p. 9). Taking a lead from the quads’ parents, researchers also downplayed the evidence of sexual and physical abuse in the young women’s histories ( Farley, 2023, p. 232. See also Cotton, 2015). Yet the Genain study deserves far greater attention for those things that have been all but forgotten in the obsession with genetics that followed it: the range of explanations and interactions it invited, and the things that might be learned from one incredibly detailed case study that are invisible when individuals with complex lives become simply statistics.

Just as Mendelian theories became popular amid political and economic concerns around degeneration and eugenics, the popularity of Genain genetics also served a purpose. While psychoanalysis had dominated in the United States in the immediate post-war period, and social psychiatry continued to influence British mental health science, two inter-related factors shifted the focus in both countries. First, both anti-psychiatry and the survivor movement in the 1960s and ‘70s emphasised a socio-environmental model of mental health, drawing on the counterculture of the 1960s to question traditional hierarchies of psychiatric treatment as well as models of causation ( Cooper, 1967; Laing & Esterson, 1964; Wall, 2019). The rise of the therapeutic community encapsulated this approach for many – these tended to be non-hierarchical spaces that focused on social and inter-personal concerns, rejecting biological aetiologies of mental ill-health ( Spandler, 2006). Alongside this, the prestige of the profession was declining. Psychiatry was negatively presented in the media, disliked by patients and families and, by 1973, subject to legal challenges, as courts began to over-rule psychiatrists’ decisions ( Decker, 2013, p. xvii). Historian Hannah S. Decker has charted how the creation of the third edition of the American Psychiatric Association’s Diagnostic and Statistical Manual (DSM-III) emerged as a response to these concerns, aiming to ‘show that psychiatry was a truly scientific discipline worthy of wide respect’ ( Decker, 2013, p. xvi).

The goal of the architects of DSM-III, led by neo-Kraepelinian Robert Spitzer, was a descriptive psychiatry. They wanted to create clear universal diagnostic guidelines, which would lead to the same patients being diagnosed in the same way across continents and cultures. According to Decker, Spitzer’s main concern was reliable diagnosis. The existential ‘reality’ of schizophrenia or any other syndrome (and its causes) mattered less to him than whether people with similar symptoms were being diagnosed in the same way ( Decker, 2013, p. 132). But, of course, universal diagnostic guidelines serve to reinforce the notion of a unitary illness: if schizophrenia is diagnosable, it therefore exists. This tautological position continues to be questioned in both medical and historical literature ( Boyle, 1990; Filer, 2019; McNally, 2016). However, DSM-III was undoubtedly influential in creating what appeared to be a secure basis for psychiatric diagnosis and research.

Because the aims of Spitzer and his colleagues were descriptive, the DSM had little to say about causation, admitting that, for most disorders, aetiology was unknown ( Force, 1980, p. 6). Its authors claimed that the DSM-III approach was to be ‘atheoretical with regard to etiology or pathophysiological process except for those disorders for which this is well established and therefore included in the definition of the disorder’. They concluded that ‘undoubtedly, with time, some of the disorders of unknown etiology will be found to have specific biological etiologies, others to have specific psychological causes, and still others to result mainly from a particular interplay of psychological, social and biological factors.’ ( Force, 1980, p. 7) There are two interesting points to make in regard these statements. One is that DSM-III continued to acknowledge a range of factors in causation, as well as the complex interplay between them. Second, certain ‘well established’ aetiologies were taken at face value and ultimately gained greater weight when incorporated into the descriptive approach of the DSM as unambiguously factual. Yet, despite claiming to be objective in its descriptions, the DSM has always been formed by the context in which it was written.

The DSM-III was adamant that there was a genetic basis for schizophrenia. This was not the case with the vast majority of other diagnoses, including Major Affective Disorders, where no specific conclusions were drawn about ‘familial patterns’ ( Force, 1980, p. 217). Based on the twin studies of the previous decades, the manual stated baldly that ‘genetic factors have been proven to be involved in the development of the illness’, while admitting that ‘the existence of a substantial discordance rate, even in monozygotic twins, indicates the importance of nongenetic factors’ ( Force, 1980, p. 186). The DSM was not the only place such claims were found. In a 1975 discussion on the genetics of schizophrenia, it was similarly claimed ‘without much doubt that a firm biological foundation for schizophrenia exists’ ( Dunham, 1975, pp. 210–1). DSM-III attempted to dispel the ongoing concern of geneticists about whether schizophrenia was a discrete, diagnosable disease, an uncertainty that implied their research might lie on shaky foundations. Thus, things came full circle. At the 1985 Biological Psychiatry conference, researchers made much of the opportunities afforded by the new DSM-III diagnosis of schizophrenia for genetic research ( World Congress of Biological Psychiatry and Shagass, 1986, pp. 52–64).

The media was also central to the growing influence of genetic explanations of mental illness, especially schizophrenia. Sociologist Peter Conrad describes 1987–1994 as a key period of ‘genetic optimism’ in the American media. Before the mid-1980s, newspapers were hesitant about making a direct link between genetics and mental illness. However, from the mid-1980s, press articles shared the common belief that a gene for a particular psychiatric disorder existed, that it would be found and that finding it would have benefits for treatment. Studies purporting a genetic link for a particular diagnosis became headline news, while disconfirmations or contradictory research were rarely reported. For example, in 1987 Egeland and colleagues found a genetic marker that they linked to manic-depressive disorder in the Old Order Amish of Pennsylvania, reported with much fanfare. Two years later, another study concluded that, following continued research into the Amish, the genetic marker was probably not significant after all. This was mentioned in passing in some newspapers, but not in science magazines. Conrad suggests that this style of reporting might serve not only to predispose lay readers towards genetic interpretations of mental ill health, but is also ‘almost certain’ to have an effect on the way journalists, scientists, and researchers view genetics and mental illness ( Conrad, 2001, p. 244).

## From human genome to gene wide association studies and epigenetics (post-1990)

The Human Genome Project (HGP) emerged from this new genetic optimism. Regarded as the start of ‘the era of the genome’ by modern geneticists ( Rutherford, 2022, p. 178), this huge scientific project aimed to read the entirety of a single person’s DNA. The project hub, at Cold Spring Harbor Laboratory, had, long before, housed the Eugenics Record Office, reminding us that scientific research into genetics is directly related to its hereditarian history ( Rutherford, 2022, p. 64). Beginning in 1990, the Human Genome Project was expected to take 15 years to complete. It came in under budget – and early – with the first draft sequence published in summer 2000, and the project fully completed in 2003 ( Harper, 2008, pp. 378–82). Of course, technologies also changed while the project was underway, not least because the project itself emphasised the development of ‘more efficient and cheaper methods of DNA sequencing’ ( Harper, 2008, p. 379).

By making genetic data available to other researchers, the Human Genome Project sparked a host of studies, many still searching for the elusive ‘schizophrenia gene’. The private company of HGP’s rival, Craig Venter, hoped to win the race to map the genome in order to patent ‘the three hundred most important genes’ ( Mukherjee, 2016, p. 312). These were described as those that could be targeted by new drugs to combat a range of diseases, including schizophrenia. Some psychiatrists hoped that the project would finally reveal all about the genetics of mental health: this was not the case. ( Porter, 2018, p. 346).

Many biological psychiatrists instead began to place their faith in a newer method of laboratory research, Genome Wide Association Studies (GWAS), introduced in 2005. GWAS involves comparing the genetic data of a large group of people with the same condition or trait against a control group to look at which areas stand out. Schizophrenia, claimed by previous generations of researchers to be the most heritable of mental disorders, is the most common diagnosis to be investigated. In 2018, for example, one European GWAS of 11,260 people diagnosed with schizophrenia (and 24,542 who were not) found 50 new sites of difference, resulting in a total of 145 DNA differences across the whole genome. This suggested that all 145 genetic variables (if not more) contributed to the likelihood of being diagnosed with schizophrenia ( Pardiñas *et al.*, 2018). A UCL study analysing Polygenic Risk Scores and Copy Number Variants (CNVs) acknowledges that the ‘predictive power’ of the single nucleotide polymorphisms associated with psychosis is, individually, ‘relatively small’ – and so the calculation of polygenic risk scores has become the priority for furthering GWAS research ( *Copy number variants and polygenic risk scores in psychotic disorders*, 2023).

The Psychiatric Genomics Consortium (PGC) was founded in 2007 to bring together researchers with an interest in genetics. Today, the PGC claims involvement from more than 800 international scientists and 900,000 participants ( PGC website, 2024), making it the largest biomedical research project in the history of psychiatry. The group’s aim is to ‘convert the family history risk factor into biologically, clinically, and therapeutically meaningful insights’ ( Sullivan *et al.*, 2018, p. 2). The founders share a positivity about psychiatric genetics that glosses over the more problematic parts of its history, assuming a clear line of progress whereby twenty-first century discoveries build on nineteenth and early twentieth century claims about inheritance ( Sullivan *et al.*, 2018, pp. 2–3). As we have shown in this review, such a teleological approach is mistaken.

The PGC has been central to the development of GWAS studies, through the group’s facilitation of a structure to bring research together, making summary statistics of all analysis openly available online ( Sullivan *et al.*, 2018, p. 5). This created a field in which the vast sample sizes necessary for GWAS studies became gradually available to its researchers, with data sharing and the world wide web thus necessary components of genetic research. Between 2011 and 2014, the PGC’s schizophrenia data sample jumped from 9,394 to more than 36,500 cases (and 113,000 controls), vastly increasing the potential to draw associations between sites of DNA difference ( O’Donovan, 2015, p. 292; Ripke *et al.*, 2014). This led to increased optimism about the potential for GWAS, with the PGC’s 2014 study finding 128 independent associations ‘that meet genome-wide significance’ ( Ripke *et al.*, 2014, p. 421).

There remains debate about the clinical utility of GWAS studies. While the PGC is clear that polygenic risk scores cannot be used to predict the likelihood of developing symptoms or the possible trajectory of illness (‘Information for Clinicians’, PGC website, 2024), in the last five years a number of researchers have attempted to develop tools to increase the clinical usefulness of GWAS research. In 2020, for example, Reay et al developed a measure they called the ‘pharmagenic enrichment score’ (PES), designed to link common gene variants to responsiveness to existing medication ( Reay *et al.*, 2020). Their aim was to offer more precise treatment options to individuals diagnosed with schizophrenia. Meanwhile, testing for rare variants (described as SNVs and CNVs) has become common in genetics laboratories, particularly CNVs. This, researchers hope, will yield greater information about the role of rare genetic variations in diagnoses of autism, schizophrenia and bipolar disorder ( Malhotra & Sebat, 2012).

Where does the field of Genome-Wide Association Studies stand today, nearly two decades after its genesis? A 2023 review article by Pergola *et al.* in *Biological Psychiatry*, one of the flagship peer-reviewed journals in the field, argued that there was a significant gap between the estimation of genotype-based heritability, and the type of heritability indicated by twin studies:

This heritability gap suggests a complex genetic architecture, a potential role of nonadditive genetics and gene-environment interplay, heterogeneous etiologies and clinical manifestations, or a combination of these factors. In short, we have started deciphering the genetic code for SCZ [schizophrenia], but many questions remain unanswered ( Pergola *et al*., 2023, p. 121).

The authors argue – with plentiful caveats – that the field’s potential now lies in ‘parsing’ the genetic risk scores that may correspond with potential biological pathways. The statistical risk score and genome-wide associations are valuable for understanding aetiology and developing therapeutics only insofar as they can point towards the potential gene-expression mechanisms that might have causal agency for specific symptoms. Some areas of exploration in this domain have included gene regulation (particularly in relation to dopaminergic and glutaminergic pathways), and cellular processes in relation to cortical thickness abnormalities. Others relate to the data on co-expression of particular genes and how these may correlate with processes relating to ‘axon development, synapse function, and hemophilic cell adhesion’ ( Pergola *et al*., 2023, p. 125). The authors conclude that the limited evidence that has been generated by these studies suggest that the odds are against an aetiological explanation for schizophrenia based on a single genetic or biological ‘signature’. Instead, ‘clusters of patients may share etiologic factors falling into different scenarios of coherence, possibly with a different balance of genetic and environmental factors at the individual level’ ( Pergola *et al.*, 2023, p. 126).

The polygenic theory of causation is today widespread, if not universal ( Pardiñas *et al.*, 2018, p. 382). Most geneticists also believe there are psychological, social, cultural and environmental factors which affect whether or not someone with a particular genetic make-up develops symptoms of schizophrenia. Indeed, there are also GWAS research programmes attempting to parse out the genetic and the environmental, with one recent paper on anxiety going so far as to claim that ‘reported life events are partly heritable’, by arguing that ‘differences in how individuals process the contextual aspects of the environment or interpret their own physical and emotional response to environmental stimuli may be one mechanism through which genetic liability influences the subjective experience of life events’ ( Peel *et al.*, 2023). No doubt psychologists of a different persuasion would have something to say about how the intergenerational transmission of sensitivity could be accounted for by non-genetic factors such as parental communication, maladaptive belief systems, or unconscious interpersonal dynamics, reminding us that explanations for inherited traits do not always rely upon genetics as the causal mechanism. Yet researchers from each of these perspectives present their conclusions in assertive terms, in line with the expectations of scientific publishing and grant capture. These bold conclusions often mask the lack of consensus within psychology or psychiatry, before we even consider mental health science in the round.

A curious consequence of the diversity of hypothesised gene-expressions and neurobiological pathways has been a movement towards a more spectrum-based understanding of schizophrenia, re-casting the diagnosis as a heterogeneous syndrome rather than a coherent disease category ( Cuthbert & Morris, 2021; Pergola *et al.*, 2023). This perspective also built upon a shift within the US National Institute for Mental Health’s research agenda from a decade earlier, with the creation of the Research Domain Criteria Initiative, recalibrating research on mental disorders around six domains of ‘dysfunction in fundamental psychological/biological systems’, using these processes as the defining criteria rather than the psychiatric diagnostic categories described in the DSM or International Classification of Disease (ICD) research manuals (2023).

There is a degree to which one of the main – and most counter-intuitive – effects of genetic research in mental health has been to destabilize one of the longest-established categories in the discipline of psychiatry: schizophrenia. By failing to establish the organic aetiological mechanisms of the most classic of the so-called organic psychoses, biologically orientated research has had to radically re-define and broaden the object of its study into multiple possible objects. The tentative, heavily-caveated, hypothesis-driven and cautious approach taken by the authors of these research papers when coming to conclusions about aetiology is marked. While there is significant optimism about the potential for benevolent outcomes from this research, and a constant refining of the most viable avenues to be pursued, there is also substantial caution in relation to the strength of the evidence as it currently stands, and the power of its predictive meaning. As Adam Rutherford points out, ‘It is perfectly possibly to have every one of those 145 genetic variants, and never show the slightest sign of schizophrenia’ ( Rutherford, 2022, p. 173).

In a landmark review of research on ‘Defining the Genetic, Genomic, Cellular and Diagnostic Architectures of Psychiatric Disorders’ in *Cell*, psychiatric geneticists Patrick Sullivan and Daniel Geschwind also tackle the implications of these lines of research for clinical genetic testing. In relation to schizophrenia, they posit that there could be value in testing where there are large copy-number variants (CNVs), which ‘in severe psychotic disorders (SCZ and schizoaffective disorder) will be present in 3%–5% of cases. The utility is diagnostic and in ameliorating medical morbidity given that most CNVs are multi-system disorders carrying additional medical risks.’ ( Sullivan & Geschwind, 2019, p. 176). They do, however, foreground the social and commercial reality of genetic testing, pointing out that governmental regulatory processes have failed in many countries, leading to consumers accessing genetic tests with a ‘weak scientific basis’. They also point out that serious discussion of the potential for testing would need to take into account the availability of technologies, national regulatory standards and – importantly – ethics ( Sullivan & Geschwind, 2019, p. 176). Given the entanglement of the history of psychiatric genetics with eugenics and euthanasia, as we have seen above, ethics must remain central to discussions of the clinical implications of psychiatric genetics, and particularly testing technologies. This point seems all the more pertinent given a noted resurgence of overt eugenic thinking in contemporary politics ( Allen, 2018; Haynes, 2020).

As Theodore Porter has shown, much of the history of psychiatric genetics is also the history of statistics ( Porter, 2018). Beginning with the introduction of the correlation table in 1860, psychiatric researchers tried a range of ways to prove their hypotheses about the hereditary nature of diagnoses. These statistical methods were themselves open to intense debate and interpretation. In the early 1900s, Wilhelm Weinberg’s research into population genetics caused him to criticise the methods of eugenicists Rosanoff and Orr, and their use of selective statistics ( Gausemeier, 2015, pp. 478–9). Others, who did utilise Weinberg’s correction methods, nonetheless continued to stand by their belief in a simple Mendelian model of inheritance ( Gausemeier, 2015, p. 479). More recently, new statistical methods such as linkage disequilibrium (LD) score regression, developed by Bulik-Sullivan *et al.*, have attempted to account for the relative contributions of genetic risk and other factors in GWAS studies ( Bulik-Sullivan *et al.*, 2015). Transcriptome-wide association studies (TWAS) were similarly developed to detect which gene-trait associations are significant ( Gusev *et al.*, 2016), while Mendelian randomisation aims to test a causal hypothesis ( Smith & Ebrahim, 2003).

New methodologies have also sought to account for bias in genetic sampling. As we can see from the history of psychiatric genetics, the vast bulk of research studies have been carried out in the UK, Western Europe and the US. This, as one recent study pointed out, carries the risk that using findings from GWAS and PRS may exacerbate health inequalities across populations. To account for this, Zhang *et al.* used data from commercial DNA testing to develop CT-SLEB, a method of calculating PRS using ‘ancestry-specific GWAS summary statistics’ ( Zhang *et al.*, 2023, p. 1757). There remain relatively few peer-reviewed multi-ancestry studies within psychiatric genetics, an important area for development. In the first such study of major depression, Meng *et al.* noted that their multi-ancestry GWAS found that many loci from European ancestry samples were unique and not transferrable to other groups ( Meng *et al.*, 2024, p. 230). They also noted the limitations of their own samples: their ancestry data was not global, and largely limited to individuals of different ancestral heritage living in the US or UK.

A further line of active, promising research has been the modern epigenetic approach, which explores ‘the effect of environmental factors on gene expression’: that is, the biochemical ways in which the environment (in the broadest sense of the word) impacts on gene activity, without the DNA sequence itself being modified. These modifications can, in turn, have inter- and transgenerational effects on offspring (with inter-generational being from one generation to the next, and trans-generational denoting an effect across three or more generations) ( Joseph *et al.*, 2013, p. 83). The impact of any genetics can be altered not only by whether or not there are mutations in a specific gene, but also by the extent to which individual genes are differentially expressed. When gene expression is regulated by changes not in the DNA code but to the structure of the DNA molecule, this is referred to as an epigenetic change. These processes have significant influence in early life and may be predictive of a range of health outcomes in later years. Researching epigenetic effects is challenging in psychiatry, however, as the neural tissues that yield the relevant data in living humans is, for obvious reasons, difficult to access. As a consequence, researchers have looked to other strategies including animal models, examination of post-mortem neural tissue samples, and blood biomarkers ( Keverne & Binder, 2020; Ryan *et al.*, 2018).

Studies have linked the glucocorticoid system to anxiety disorders and PTSD, and potentially to intergenerational transmission of trauma in the families of Holocaust survivors ( de Quervain *et al.*, 2017; Yehuda *et al.*, 2014). Micro-RNAs (miRNAs) are small non-coding RNA molecules which are involved in epigenetic gene expression regulation, which are found in high concentrations in brain tissue. They have been implicated in depression, potentially in processes relating to dysregulation of monoamines and inflammatory responses, and in relation to neuroplasticity ( Ding *et al.*, 2023; Ryan *et al.*, 2018; Roy *et al.*, 2017). Beyond miRNA, a good deal of contemporary research in psychiatric epigenetics is linked to a process called DNA methylation, involving the addition of a methyl, or CH3 group onto the DNA’s cytosine or histone proteins ( Keverne & Binder, 2020). Methylation was causally linked to schizophrenia by a number of researchers in the mid-2010s, with research still continuing along this path ( Jaffe *et al.*, 2016; Mitchell *et al.*, 2018; Viana *et al.*, 2017). More recently, it has been hypothesised that dysfunction of the N-methyl D-aspartame Receptor (NMDAR), which is a key mediator of synaptic plasticity, is implicated in both schizophrenia and depression ( Flynn & Gao, 2023; Richetto & Meyer, 2021). Further research into the therapeutic activity of psychedelic substances in mental health has hypothesised that DNA methylation could be key to their mechanism of action and may play a role in reversing the effects of NMDAR dysregulation in depression, with potential therapeutic implications for the treatment of schizophrenia as well ( Flynn & Gao, 2023). It is important to reiterate that epigenetic research is in its infancy, is tentative, and that the techniques available to test hypotheses are far from uncomplicated.

The modern epigenetic approach still tends to focus on heredity and biological processes first and environment second. We do not know for certain what variations in genetic code mean, what they do, or how they interact with each other and external factors, such as trauma and socio-cultural experiences. This means that epigenetics has, as yet, resulted in little more in the way of practical applications in mental health than its predecessors, although a good deal of theorization is underway to connect theory to mechanism, which may eventually have implications for practice. As Happe noted, epigenetics ‘presumes the individual to be the site of environmental health interaction’, with a resulting focus on individual behaviour modification, or pharmaceutical treatment, rather than regulation of environmental factors ( Happe, 2013, p. 21). ‘Nevertheless, I had to wonder’, she concludes in one evocative example, ‘do we really need to know how gene variants predispose someone to have an adverse response to cockroach droppings? Shouldn’t we just get rid of the cockroaches?’ ( Happe, 2013, p. 139).

It surprised the authors of this paper that our small survey involving the insights of people with lived and living experience highlighted genetics and heredity as important factors through which people with a diagnosis of mental ill-health understand their experiences. As one anonymous respondent put it, ‘I have experienced that biological approaches (e.g. medication) have helped me more than I would have thought possible, which has made me realise that a diathesis-stress model of genetics/environment makes a lot of sense to me.’ Others listed genetics, heredity or intergenerational trauma as a key factor in their own mental health, though often as part of a long list of potential causes. This did not make them uncritical of diagnosis, treatment and other aspects of the mental health system.

This is unlike the survivor movement of the 1980s and 1990s, which rejected a genetic approach to mental distress entirely ( Marks *et al.*, 2023, pp. 4–5). Our review of every issue of *Asylum*: *the radical mental health magazine* between 2014 and 2023 found only 3 articles that mentioned genetics, two of which were critical reviews of science reporting. However, the third article perhaps more closely relates to some of the lived and living experience perspectives from our survey. Joel Petrie’s ‘The Merry Men of My Family’ (2021) commented on a long history of male ‘eccentricity’ in his family. Petrie nonetheless concluded that: ‘Politically, I am firmly in the social model of disability camp; and recognise that the notion of a genetic component to madness can be controversial. It is without doubt the case that most distress in terms of “mental health” is a consequence of social and political factors’ ( Petrie, 2021, p. 7). David Cotton, the son of the only Genain quad to have children of her own, similarly expressed surprise at the obsession of NIMH researchers with the brains and biology of his family, and their lack of interest in his life experiences ( Farley, 2023, p. 197). To Cotton, these experiences were far more relevant to his own mental health history. While genetics has become incorporated into medical *and* lived experience accounts of causation in mental ill-health, the ‘precipitating factors’ that result in outright illness may be given more weight by those with lived experience than by mental health scientists.

## Conclusion

This history of heredity and genetics in mental health causation illustrates a number of overall trends. The first, and perhaps most important, is the interplay between assumption and evidence. From the birth of the asylum, and perhaps even before, doctors believed heredity to be a factor in the existence of mental illness. Many of them found evidence because it was what they were looking for – completely unspecific odd behaviour in relatives, for example. While acknowledging this does not prove that heredity is *un*important in mental health aetiology, it has contributed to a lack of interest in following up other avenues of research that might be equally fruitful. When research did not support pre-existing beliefs – as in the case of Rüdin’s assumption that schizophrenia was inherited as a single-gene Mendelian trait – some scientists performed explanatory gymnastics in order to retain the same or a similar model of inheritance. In some cases, this research functioned to legitimize social and political agendas, such as eugenics.

The notion of Mendelian traits highlights another complicating factor: *what* exactly do we think we are inheriting? From the nineteenth century to the late 1970s, psychiatrists were uncertain whether psychiatric diagnoses met the criteria for discrete diseases and, later, ‘unit factors’ in Mendelian inheritance. The notion of ‘neurotic inheritance’ emerged as a way of countering this complication, alongside the idea that an ‘exciting’ factor might be needed to tip someone into outright disease. From 1980, however, the popularity of DSM-III gave psychiatric diagnosis a certainty it had not previously enjoyed, sparking further efforts to find a ‘schizophrenia gene’. Today, even with interest in epigenetics widespread, some scientists still assume that, when they look for genetic markers for schizophrenia they are studying one discrete condition ( Mukherjee, 2016, p. 442; Rutherford, 2022, p. 173). But can we be sure that the thousands of cases in a Genome Wide Association Study are, in fact, instances of the exact same thing? Contemporary shifts in psychiatric genetic research published in key journals such as *Biological Psychiatry* and *Frontiers in Psychiatry* indicate a move away from this long-standing consensus, and that schizophrenia, one of the most stable categories since the birth of modern psychiatry, may be being disassembled from within the field itself ( Cuthbert & Morris, 2021; Pergola *et al.*, 2023).

While monogenic, biological approaches have had their advocates (most notably Eliott Slater), today, most studies on the genetics of mental ill health tend towards a polygenic, multifactorial approach. However, it is the biological aspect of this that has received the most attention, from the Genain study onwards, with far less research into environment and interaction. While this gap was noted in the 1960s and 1970s ( Dunham, 1975, p. 213; Rosenthal, 1963, p. 575), there does not appear to have been as much attention paid to it since. As Read and Sanders noted, a 2008 analysis found that ‘for every study into the social causes of psychosis, there had been 15 into biological factors’ ( Read & Sanders, 2022, p. 29). Has this history of emphasis and exclusion helped or hindered the search for clear aetiologies of mental illness? We are, after all, only just beginning to appreciate how the emphasis on data gathered in Western European and North American countries has impacted findings on sites of genetic difference.

There are a number of reasons for this focus on genetics and inheritance in mental health aetiology, and schizophrenia in particular, which have been outlined in this paper. As psychiatry struggled to maintain its relevance in the 1960s and 1970s, genetics appeared to promise a more secure biological footing for understanding causation. Many researchers, especially following the emergence of the Human Genome Project, hoped that a genetic basis for mental diagnoses would lead to improved treatment. But has that optimism been well founded? Harper claims that psychiatric genetics remained separated from medical genetics in the twenty-first century largely because there ‘is no immediate prospect of predictive tests or other genetic applications’ within psychiatry ( Harper, 2008, p. 338). What, then, *is* the treatment for ‘faulty’ genes?

This highlights the biggest concern surrounding genetic theories of schizophrenia, echoed in recent review articles in *Biological Psychiatry* and *Cell*: if we do not know *why* certain genetic factors put someone at risk of developing symptoms, what do we do with our knowledge about factors that seem to be relevant? From the 1880s through to the 1950s, most psychiatrists would have assumed this knowledge to have a eugenic benefit, which focused on the economic and political ramifications of their research. While there may yet be potential for both greater aetiological understanding and therapeutic benefit, with new statistical approaches attempting to better target pharmaceutical therapies, a focus on genetic aetiologies of mental illness thus raises significant ethical concerns. James Watson, the first director of the Human Genome Project, recognised this, allocating 3% of his budget to humanities scholars to study the social, ethical and legal implications of the research: an important factor in building public trust in the project ( Harper, 2008, p. 379). However, it is the scientific outcomes of the project – largely divorced from this social and ethical context – that remain in use today, with much of the surrounding research forgotten.

What do we do with the knowledge that at least 145 genetic markers are associated with the risk of being diagnosed with schizophrenia? Certainly, there are promising avenues for further research. This might include identifying new therapeutic agents. In terms of treatment and prevention, however, we might also consider what Victorian psychiatrists would have called ‘exciting factors’ in mental health. After all, the hereditary condition of phenylketonuria in infants, which leads to significant mental symptoms if ignored, is treated primarily through environmental intervention: a low-protein diet. We could spend more time on trying to understand gene-environment interaction, on exploring environmental factors that might cure or alleviate a particular condition, and on ensuring – through interdisciplinary projects – that mental health science is contextualised within the society in which it is utilised. This, it seems, would fit most closely into the expectations of those with lived experience of psychosis; although this expectation in itself is worthy of further study.

## Data Availability

The source data for this article which consisted of bibliographic references, are included in the References section. The lived experience survey and focus group data cannot be made accessible, since these sessions were carried out on the basis that only anonymous quotations be used and, in the case of the focus groups, that these would be agreed by participants before publication. Ethics approval was given on the basis that: “We do not intend to share raw data with the Wellcome Trust.” The survey was not data underpinning the research, but a review of lived experience that raised additional questions as outlined in the methodology statement. As such the raw data is unnecessary for anyone wishing to replicate or continue this research.
