# Supplementary material for: ‘Almost nothing is firmly established’: A History of Heredity and Genetics in Mental Health Science
Source: Wellcome Open Res. 2024 Aug 8;9:208. Originally published 2024 Apr 18. [Version 2] doi: 10.12688/wellcomeopenres.20628.2 (PMC11362721; doi:10.12688/wellcomeopenres.20628.2)
Supplement: Peer review history

# Reviewer report: Reviewer response for version 2

**DOI:** 10.21956/wellcomeopenres.25230.r92692

**Published:** 2024-10-10

**Authors:** Roberta Passiatore

**Affiliations:** 1 Lieber Institute for Brain Development, Baltimore, Maryland, USA

I am satisfied with the revisions made by the authors in response to the feedback received. I have no further concerns.

 As a minor note, I recommend adding the page number to the reference (Slater, 1971, p. xx).

Is the work clearly and accurately presented and does it cite the current literature?

Yes

If applicable, is the statistical analysis and its interpretation appropriate?

Not applicable

Are all the source data underlying the results available to ensure full reproducibility?

No source data required

Is the study design appropriate and is the work technically sound?

Partly

Are the conclusions drawn adequately supported by the results?

Yes

Are sufficient details of methods and analysis provided to allow replication by others?

Partly

Reviewer Expertise:

Neuroimaging, imaging-genetics, computational genetics, schizophrenia, psychotic disorders, cognition, risk prediction

I confirm that I have read this submission and believe that I have an appropriate level of expertise to confirm that it is of an acceptable scientific standard.

# Reviewer report: Reviewer response for version 2

**DOI:** 10.21956/wellcomeopenres.25230.r92693

**Published:** 2024-08-29

**Authors:** Dylan Kiltschewskij

**Affiliations:** 1 University of Newcastle, Newcastle, Australia

I am satisfied with the author's response to the reviewer's comments in this revised article.

Is the work clearly and accurately presented and does it cite the current literature?

Yes

If applicable, is the statistical analysis and its interpretation appropriate?

Not applicable

Are all the source data underlying the results available to ensure full reproducibility?

No source data required

Is the study design appropriate and is the work technically sound?

Yes

Are the conclusions drawn adequately supported by the results?

Yes

Are sufficient details of methods and analysis provided to allow replication by others?

Yes

Reviewer Expertise:

Complex disorder genomics, epigenetics, statistical genetics, gene expression, psychiatric illness, molecular heterogeneity.

I confirm that I have read this submission and believe that I have an appropriate level of expertise to confirm that it is of an acceptable scientific standard.

# Reviewer report: Reviewer response for version 1

**DOI:** 10.21956/wellcomeopenres.22831.r83013

**Published:** 2024-08-26

**Authors:** Brenda Cabrera Mendoza

**Affiliations:** 1 Yale School of Medicine, West Haven, USA

Chaney et al. performed a comprehensive review on the history of psychiatric genetics, with a primary focus on schizophrenia. The manuscript is well-organized chronologically, providing a relevant historical context and highlighting significant milestones in the field. However, I have a few recommendations to improve the manuscript: 

1. Please provide more details regarding the literature sources used, including the keywords and the timeframe of the literature search.  
2. Include additional details about the lived experience survey and focus groups. Specifically, mention the timeframe during which the survey was administered, the selection process for participants, and the content of the survey.  
3. It would be pertinent to discuss the increases in sample size that have occurred in genome-wide association studies (GWAS) of psychiatric disorders. Highlighting these increases is important because they enhance statistical power and contribute to the discovery of new loci. 
4. I suggest including a discussion on the efforts to increase ancestry diversity among participants in psychiatric genetic studies. Additionally, mention the rise of multi-omic studies that, when combined with genetic data might provide biological insights into mental disorders.  

Is the work clearly and accurately presented and does it cite the current literature?

Yes

If applicable, is the statistical analysis and its interpretation appropriate?

Not applicable

Are all the source data underlying the results available to ensure full reproducibility?

Yes

Is the study design appropriate and is the work technically sound?

Yes

Are the conclusions drawn adequately supported by the results?

Yes

Are sufficient details of methods and analysis provided to allow replication by others?

Yes

Reviewer Expertise:

Genetics; Psychiatry; Suicide

I confirm that I have read this submission and believe that I have an appropriate level of expertise to confirm that it is of an acceptable scientific standard, however I have significant reservations, as outlined above.

# Reviewer report: Reviewer response for version 1

**DOI:** 10.21956/wellcomeopenres.22831.r84593

**Published:** 2024-06-21

**Authors:** Roberta Passiatore

**Affiliations:** 1 Lieber Institute for Brain Development, Baltimore, Maryland, USA

Chaney et al. present an intriguing review of the heritable aspects of psychiatric disorders, tracing their research history over the past century and highlighting their significance in researching the causative mechanisms of psychiatric conditions. The authors efficiently synthesize a century of models for psychiatric disorders, with a particular focus on schizophrenia, providing a mostly historical overview of the most significant milestones.

 However, there is a significant concern that, despite the thorough historical overview, the authors’ point of view on the subject is unclear. The review only partly explains how a century of research, models, and perspectives can guide future research in the field of schizophrenia and other psychiatric conditions. This is a crucial point for the review's utility.

 Additionally, several formal and content-related points require further attention:

 1. The methods section does not specify how the literature review was conducted, including the consulted sites (e.g., PubMed), the keywords used to extract the literature discussed in the text, and the archival material consulted at the Bethlem Museum of the Mind.

 2. The objectives and procedures used in the focus group and the subsequent survey need to be explained or referenced. The authors also need to clarify survey results and how they intend to integrate them in the review. Clarifications on this matter are necessary.

 3. Regarding the information retrieved from hospital records from the early 1900s, it would be better to discuss "familiality" for the disorder when collecting patient history rather than using the term "heritability." The term "familiality" is more appropriate as it includes both heritable and environmental aspects, which are essential to discuss in this context. It would also be essential to ask if the model of causation considered both genetics and the familial environment since the early 1900s, and how models changed over time.

 4. The authors mention the impact of recent Genome-Wide Association Studies (GWAS) but do not reference the Psychiatric Genomics Consortium, which played a crucial role since 2007 in discovering schizophrenia-associated loci. Major findings and the latest updates should be reported.

 5. Exploring the causal mechanisms and genetic risk factors of schizophrenia and other disorders has required and still requires methodological and statistical advancements. The authors could discuss these considerations by analyzing the methodological challenges for future research.

 6. As the introduction acknowledges, many studies have been conducted in Europe and North America, presenting an undeniable bias regarding ancestry. To complete the review, it would be important to mention the recent perspectives on multi-ancestry risk prediction and/or the commonalities and differences in genetic findings across ancestries.

 7. The authors did not report the focus group and/or survey results mentioned in the methods section.

 Minor comments:

 - Some references are not indexed (e.g., Porter on page 5).

 - A considerable portion of the review is dedicated to historical aspects in a didactic form. The authors might consider summarizing some historical passages in favor of more recent discoveries that could have greater relevance and utility in depicting the current state of the art and guiding future research.

Is the work clearly and accurately presented and does it cite the current literature?

Yes

If applicable, is the statistical analysis and its interpretation appropriate?

Not applicable

Are all the source data underlying the results available to ensure full reproducibility?

No source data required

Is the study design appropriate and is the work technically sound?

Partly

Are the conclusions drawn adequately supported by the results?

Yes

Are sufficient details of methods and analysis provided to allow replication by others?

Partly

Reviewer Expertise:

Neuroimaging, imaging-genetics, computational genetics, schizophrenia, psychotic disorders, cognition, risk prediction

I confirm that I have read this submission and believe that I have an appropriate level of expertise to confirm that it is of an acceptable scientific standard, however I have significant reservations, as outlined above.

# Reviewer report: Reviewer response for version 1

**DOI:** 10.21956/wellcomeopenres.22831.r84588

**Published:** 2024-06-08

**Authors:** Dylan Kiltschewskij

**Affiliations:** 1 University of Newcastle, Newcastle, Australia

In the present article, Chaney *et al *provide a comprehensive review of the history of psychiatric genetics, ranging from 19 ^th^ century asylum psychiatry to modern day genome-wide association studies (GWAS). The manuscript particularly focusses on the familial inheritance associated with schizophrenia and explores how these early findings developed over time in conjunction with Mendelian inheritance, monogenic and polygenic theories of schizophrenia, the Human Genome Project, GWAS, revised diagnostic criteria and the interplay with environmental factors. Overall, I find this manuscript to be a well-written, highly interesting summary of this field, that provides a timely contextualisation in light of the recent explosion of genetic information in this area. There are, however, some points I believe need to be addressed prior to indexing of this work, particularly in relation to modern day psychiatric genetics.

**Major comments**

1. The authors fail to discuss the foundation of the Psychiatric Genomics Consortium (PGC) in 2007, which represents a major milestone in the history of psychiatric genetics. Indeed, this could be an opportune place to briefly discuss some of the key findings from this consortium – such as the discovery of 108 genomic loci associated with schizophrenia in 2014 – that greatly improved our understanding of the polygenic nature of this disorder. Furthermore, it would be worthwhile discussing some major caveats from this work, including the difficulty associated with interpreting some associated loci with respect to causal mechanisms and clinical practice.
2. The authors outline the importance of utilising polygenic risk scores to capture the combined effect of many GWAS risk variants with individually small effect sizes. The authors then (correctly, in my opinion), state that the next challenge lies in unpacking the potential biological pathways underlying these scores. In my opinion, this offers a key opportunity to briefly discuss methods such as the pharmagenic enrichment score (PMID: 31964963, PMID: 36055211, PMID: 34302145), wherein polygenic risk scores for conditions such as schizophrenia are partitioned into pharmacologically actionable pathways that may inform an individual’s unique combination of genetic risk factors with potential clinical significance. Emerging methods such as this demonstrate how modern psychiatric genetics is seeking to link personalised genetic risk with precision medicines.
3. Another aspect of psychiatric genetics that may warrant brief discussion is rare variants, which have received increasing focus as whole genome sequencing has become more accessible and affordable. The discovery of some large effect-size rare, ultra-rare and singleton variants could be important for demonstrating that, in some individuals, genetic risk is not broadly polygenic, but rather, may be confined to a small subset of genes.
4. Comprehensive mining of GWAS data is foundational to better understanding the genetic basis of psychiatric illness beyond association signals. As such, I believe it is also worth briefly outlining the rise of statistical methods that seek to uncover causal mechanisms and novel risk factors using GWAS, such as linkage disequilibrium score regression, transcriptome-wide association studies and Mendelian randomisation. Indeed, such methods are also proving increasingly important for exploring the role of peripheral tissues in psychiatric illness and non-psychiatric comorbidities (e.g. cardiovascular disease) that represent a major health burden in this population.
5. Given that authors have presented a vast body of work spanning multiple centuries, the authors might consider summarising some of the key historical milestones of psychiatric genetics in a timeline-style figure or table.

Is the work clearly and accurately presented and does it cite the current literature?

Yes

If applicable, is the statistical analysis and its interpretation appropriate?

Not applicable

Are all the source data underlying the results available to ensure full reproducibility?

No source data required

Is the study design appropriate and is the work technically sound?

Yes

Are the conclusions drawn adequately supported by the results?

Yes

Are sufficient details of methods and analysis provided to allow replication by others?

Yes

Reviewer Expertise:

Complex disorder genomics, epigenetics, statistical genetics, gene expression, psychiatric illness, molecular heterogeneity.

I confirm that I have read this submission and believe that I have an appropriate level of expertise to confirm that it is of an acceptable scientific standard, however I have significant reservations, as outlined above.
